# Early insulin initiation in type 2 diabetes: efficacy, safety, and clinical implications: a systematic review with narrative synthesis

**DOI:** 10.3389/fendo.2026.1843227

**Published:** 2026-08-31

**Authors:** Osama Albasheer, Amal H. Mohamed, Nagla Abdalghani, Areej Hamdi, Doaa Abdulwahab Mohammed Ayish, Fatma Ayish, Ayyub Alssum, Ali Yahya Maashi, Lamyaa A. M. El Hassan, Tahani Madkhali, Mohammed Muqri, Aisha A. Awaf, Hatim Alessa, Afnan Madkhali, Afaf Hakami

**Affiliations:** 1 Department of Family and Community Medicine, College of Medicine, Jazan University, Jazan, Saudi Arabia; 2 Department of Internal Medicine, College of Medicine, Jazan University, Jazan, Saudi Arabia; 3 Respiratory Therapy Department, Faculty of Applied Medical Science, Jazan University, Jazan, Jazan, Saudi Arabia; 4 Department of Family Medicine, Diabetic Center, King Fahad Central Hospital, Ministry of Health, Jazan, Saudi Arabia; 5 Department of Internal Medicine, Medical and Rheumatology Consultant, Head of Medical Department, Jazan General Hospital, Jazan, Saudi Arabia; 6 Family Medicine, Primary Healthcare, Ministry of Health, Jazan, Saudi Arabia; 7 Department of Family Medicine, Jazan University Hospital, Jazan University, Jazan, Saudi Arabia; 8 Department of Pathology, College of Medicine, Jazan University, Jazan, Saudi Arabia; 9 Department of Internal Medicine, Medical Consultant, Jazan Military Hospital, Jazan, Saudi Arabia

**Keywords:** cardiovascular diseases, glycemic control, hypoglycemia, insulin therapy, systematic review, β-cell function

## Abstract

**Aims/background:**

Early insulin initiation in type 2 diabetes mellitus (T2DM) is proposed to improve glycemic control, preserve β-cell function, and potentially reduce long-term complications. Evidence suggests that starting insulin within five years of diagnosis may offer additional metabolic benefits, but its clinical impact remains uncertain. This systematic review aimed to evaluate the efficacy, safety, and challenges of early insulin use in T2DM.

**Methods:**

We systematically searched PubMed, Cochrane Library, Scopus, and Controlled Trials Register for studies published from 2010 to 2024, including adults with T2DM who initiated insulin within five years of diagnosis. Sixteen studies met inclusion criteria (9 RCTs, 7 observational), totaling 44, 045 participants. Two reviewers independently extracted data on glycemic outcomes, adverse events, and long-term complications. Risk of bias was assessed using the Cochrane tool for RCTs and the JBI checklist for observational studies. Due to substantial clinical and methodological heterogeneity, a meta-analysis was not performed, and findings were synthesized narratively.

**Results:**

Early insulin therapy significantly reduced HbA1c (by 0.8–2.3%, p<0.05) and fasting glucose (by 1.2–2.4 mmol/L). Hypoglycemia occurred in 8–15% of insulin users compared to 3–10% with oral agents; severe episodes were rare (<2%). Weight gain over 2 kg was reported in nearly half of the studies.

**Conclusion:**

Early insulin initiation improves glycemic outcomes and may preserve β-cell function. However, increased risks of hypoglycemia and weight gain warrant individualized treatment decisions. Future studies should adopt standardized definitions and assess long-term cardiovascular outcomes.

**Systematic review registration:**

https://www.crd.york.ac.uk/prospero/, identifier CRD42024508356.

## 1 Introduction

Managing type 2 diabetes mellitus (T2DM) involves both behavioral and therapeutic challenges, especially in determining the optimal timing for insulin initiation. Despite its benefits, many patients and healthcare providers hesitate to initiate insulin due to concerns about hypoglycemia, weight gain, and regimen complexity (1). Furthermore, unfamiliarity with insulin options and time constraints hinders its initiation and intensification.

Although oral hypoglycemic agents, such as metformin, sulfonylureas, and sodium-glucose co-transporter-2 (SGLT2) inhibitors, have improved diabetes management, many individuals with T2DM struggle to achieve adequate glycemic control (2). Compared to early insulin initiation, these agents may not provide sufficient β-cell preservation and often lead to progressive treatment intensification over time (3). While current international guidelines, including The American Diabetes Association (ADA) and the European Association for the Study of Diabetes (EASD), recommend initiating insulin in newly diagnosed patients with severe hyperglycemia (HbA1c ≥9–10% or fasting glucose ≥300 mg/dL) or in those presenting with catabolic features such as weight loss or ketosis, they do not provide a universally accepted temporal definition of “early” insulin initiation (4, 5). In contrast, most clinical trials and systematic reviews have operationalized “early” as insulin therapy initiated within the first five years after diagnosis of T2DM (6, 7).

Early insulin therapy contributes to vascular protection mainly through sustained glycemic control, which prevents complications. In addition, some evidence suggests potential anti-inflammatory and antioxidant benefits that may further support vascular health (2, 8). Hyperglycemia in T2DM contributes to β-cell dysfunction, exacerbated by insulin resistance and metabolic disturbances like glucotoxicity and lipotoxicity (9). Preserving β-cell function is a key therapeutic goal (2).

Clinical trials, including the Veterans Affairs Diabetes Trial (VADT), Action in Diabetes and Vascular Disease—Preterax and Diamicron Modified Release Controlled Evaluation (ADVANCE), and Action to Control Cardiovascular Risk in Diabetes (ACCORD), have shown that intensive glycemic control significantly reduces HbA1c levels compared to standard care (10–12).

Additionally, continuous subcutaneous insulin infusion (CSII) and multiple daily injections (MDI) have demonstrated efficacy in achieving normoglycemia in newly diagnosed T2DM patients (13–15). In one study (16), prevailing insulin regimens demonstrated greater efficacy in achieving glycemic control compared to prandial or premixed regimens, a finding consistent with results from the Treating-to-Target in Type 2 Diabetes (4-T) study (17), which showed that basal insulin regimens were more effective than prandial or premixed approaches in improving glycemic outcomes. Early insulin initiation, particularly with insulin glargine, has been associated with greater HbA1c reductions and a lower risk of hypoglycemia (18).

Despite evidence supporting early insulin therapy, barriers persist, including concerns about hypoglycemia, weight gain, and patient adherence, particularly among older adults, individuals with multiple comorbidities, and those in resource-limited healthcare settings where insulin education and access to newer formulations may be limited (2). Addressing these barriers is crucial for optimizing treatment outcomes in T2DM. Although insulin therapy is sometimes perceived as detrimental to quality of life (QoL), evidence suggests that early initiation does not adversely affect QoL (8, 19). In fact, short-term intensive insulin therapy has been shown to enhance diabetes-related QoL indicators, including increased treatment satisfaction (20). Moreover, the use of insulin pumps has been associated with significant improvements in QoL, contributing to enhanced self-esteem, reduced stress levels, and improved mood (21).

This systematic review examines the benefits and safety of early insulin therapy in T2DM, focusing on glycemic control, vascular health, β-cell function, and quality of life while addressing potential risks such as hypoglycemia and weight gain.

## 2 Methodology

### 2.1 Study design

This systematic review was conducted following the Preferred Reporting Items for Systematic Reviews and Meta-Analyses (PRISMA) guidelines (22). The primary objective was to evaluate the beneficial effects, safety profile, and barriers associated with the early initiation of insulin therapy in patients with type 2 diabetes mellitus (T2DM) compared to delayed initiation or alternative pharmacological therapies. For the purpose of this review, early insulin initiation was operationally defined as insulin therapy started within five years of diagnosis, consistent with prior systematic reviews and clinical trials (6, 7). As autoimmune antibody testing (to exclude latent autoimmune diabetes in adults, LADA) was not uniformly reported in the included studies, our definition was based solely on duration of diabetes rather than autoantibody status.

The primary outcomes of interest included glycemic control (HbA1c levels), hypoglycemia incidence, weight changes, and long-term complications such as cardiovascular events and microvascular complications. These outcomes were analyzed with equal emphasis.

### 2.2 Literature search strategy

A comprehensive search was performed across: PubMed (https://pubmed.ncbi.nlm.nih.gov/), Cochrane Library (https://www.cochranelibrary.com/), Scopus (https://www.scopus.com/), and Controlled Trials Register (https://clinicaltrials.gov/). The search included studies published from 2010 to 2024. The year 2010 was selected as the starting point because it marks the widespread introduction of modern basal insulin analogs (e.g., insulin glargine U300, detemir), updated international diabetes guidelines, and contemporary therapeutic philosophies emphasizing early glycemic optimization. Restricting the search to 2010–2024 ensured the inclusion of studies reflecting current standards of care and avoided outdated insulin formulations that are no longer clinically relevant. The search strategy used a combination of keywords and Medical Subject Headings (MeSH) terms, including: “Insulin therapy”, “Early initiation”, “Type 2 Diabetes Mellitus”, “Glycemic control”, “Safety”, “T2DM outcomes.” The selection of keywords was guided by previous systematic reviews, expert consultation, and preliminary searches to assess their efficacy in retrieving relevant studies. This ensured the inclusion of comprehensive and high-quality literature. The search strategy and full search strings are detailed in (**Supplementary Table 1**).

The search was conducted by two independent reviewers (one with expertise in endocrinology and the other in systematic review methodology) to ensure completeness and accuracy. Grey literature, conference abstracts, and clinical trial registries were also screened to identify unpublished studies.

### 2.3 Study selection

#### 2.3.1 Inclusion criteria

All titles and abstracts were screened by two independent reviewers. Studies were included, regardless of access restrictions, as long as they:

- Involved adult patients (≥18 years) with a diagnosis of T2DM.
- Reported outcomes related to early insulin initiation (defined as within the first 5 years of T2DM diagnosis).
- Compared early insulin initiation to delayed initiation or other pharmacological treatments (such as oral hypoglycemic agents).
- Reported outcomes including glycemic control, HbA1c levels, hypoglycemia risk, weight changes, and long-term complications (e.g., cardiovascular events, renal function).
- Included randomized controlled trials (RCTs), cohort studies, and meta-analyses of RCTs.

#### 2.3.2 Exclusion criteria

- Studies involving type 1 diabetes or gestational diabetes.
- Studies without a clear comparison between early and delayed insulin initiation.
- Case reports and reviews without original data.
- Studies not published in English due to feasibility constraints in translation.
- Observational studies with incomplete data, particularly those lacking a clear comparator group or robust methodological quality.

### 2.4 Data extraction

Data were extracted independently by two reviewers using a pre-piloted form to ensure consistency. The following data were extracted from each included study:

- Study characteristics: Author, year, country, study design, sample size, follow-up duration.
- Patient characteristics: Age, gender, duration of diabetes, comorbidities.
- Intervention: Timing of insulin initiation, dosage, combination with other treatments.
- Outcomes: Glycemic control (HbA1c levels), hypoglycemia events, weight changes, safety outcomes (including adverse effects), and long-term complications (such as cardiovascular and renal outcomes).

Using a pre-piloted form and the guidance provided by the “Cochrane Handbook for Systematic Reviews of Interventions, ” (23), disagreements were resolved by consensus or consultation with a third reviewer.

The primary research questions and objectives are mapped to the corresponding extracted variables as summarized in (**Supplementary Table 2**). This is organized according to the primary objectives and extraction domains mentioned in the introduction, data extraction, and systematic review methodologies sections.

### 2.5 Data synthesis and management of heterogeneity

Given the substantial clinical and methodological heterogeneity among included studies, a quantitative meta-analysis was not feasible. The studies differed substantially in several key domains, including population characteristics, definitions of “early insulin initiation, ” baseline glycemic status, insulin regimens used, comparator therapies, outcome definitions, and follow-up durations. Additionally, the extracted efficacy and safety outcomes were reported using different statistical metrics (e.g., absolute HbA1c change, percentage reduction, categorical response), which further limited numerical pooling. Measures of β-cell function, rates of hypoglycemia, and long-term complications also demonstrated inconsistent operational definitions across studies. Because these methodological and clinical inconsistencies violated the assumptions required for statistical pooling, the evidence was synthesized narratively, focusing on patterns, directionality, and consistency of findings rather than pooled effect estimates.

### 2.6 Statistical analysis and synthesis

A structured narrative synthesis was conducted, incorporating key principles of the Synthesis Without Meta-analysis (SWiM) reporting guideline, by collecting and summarizing the key characteristics, interventions, and outcomes reported in the included studies. We created structured tables outlining each study’s design, participant characteristics, duration of diabetes, intervention details (type, dosage, timing of insulin initiation), and primary outcomes (e.g., HbA1c, hypoglycemia, weight changes, micro- and macrovascular complications). Studies were grouped by similar outcome domains (e.g., glycemic control, safety profile, long-term complications) and qualitatively assessed whether their findings aligned or diverged. We examined any patterns of variation, such as differences in study design (RCT vs. observational), follow-up duration, measurement of outcomes (HbA1c cutoffs, definitions of hypoglycemia), and baseline patient characteristics (age, comorbidities, duration of diabetes).

### 2.7 Risk of bias assessment

Risk of bias was assessed using two validated tools. For randomized controlled trials, we applied the Cochrane Risk of Bias 2 (RoB 2) tool, evaluating domains such as random sequence generation, allocation concealment, blinding of participants and outcome assessors, completeness of outcome data, selective reporting, and other potential sources of bias. For observational studies, we used the Joanna Briggs Institute (JBI’s) critical appraisal tool (24), which assesses methodological quality across nine criteria, including clarity of inclusion criteria, validity of exposure measurement, confounding control, and completeness of follow-up. Studies scoring fewer than 4 out of 9 on the JBI checklist were considered high risk and excluded. These procedures ensured that only studies with adequate methodological rigor were included in the review.

### 2.8 Quality of evidence

The quality of evidence for each outcome was evaluated using the GRADE approach (Grading of Recommendations, Assessment, Development, and Evaluation). The certainty of evidence was rated as high, moderate, low, or very low based on: risk of bias, inconsistency, indirectness, imprecision, and publication bias (25).

### 2.9 Ethical considerations

Since this is a systematic review with narrative synthesis based on previously published studies, no new data were collected from human participants, and therefore no formal ethical approval was required. All data included in this review were derived from studies that had obtained ethical approval and informed consent from participants in their original reports. The review process adhered to principles of research integrity and transparency, including comprehensive literature search, unbiased data extraction, and accurate reporting of results, while avoiding selective reporting and conflicts of interest.

## 3 Results

### 3.1 Study selection and screening process

For the literature review, an overall of 5380 publications were retrieved, through systematic search based on (PRISMA) Guidelines. Records identified from main four databases that include PubMed (n = 5229), Cochrane (n = 114), Controlled Trials Register (n=21) and Scopus (n=19). Further articles were found by manual-searching and looking up of references of identified research (n = 4).

Rayyan online tool (https://rayyan.ai/) and manual search were used to assess for duplication, and (n=5036) duplicated articles were excluded. For the remaining identified research (n=339 articles) Automated filters (including study design, publication year, and age group) were applied to refine the search to the relevant articles that met the inclusion criteria, by which a total of 246 papers were excluded. Screening and assessment for title and abstract was conducted for (n=93 research) and accordingly another 69 articles were eliminated, (n=51) of them by title and (n=18) by abstract evaluation.

**Figure 1:**
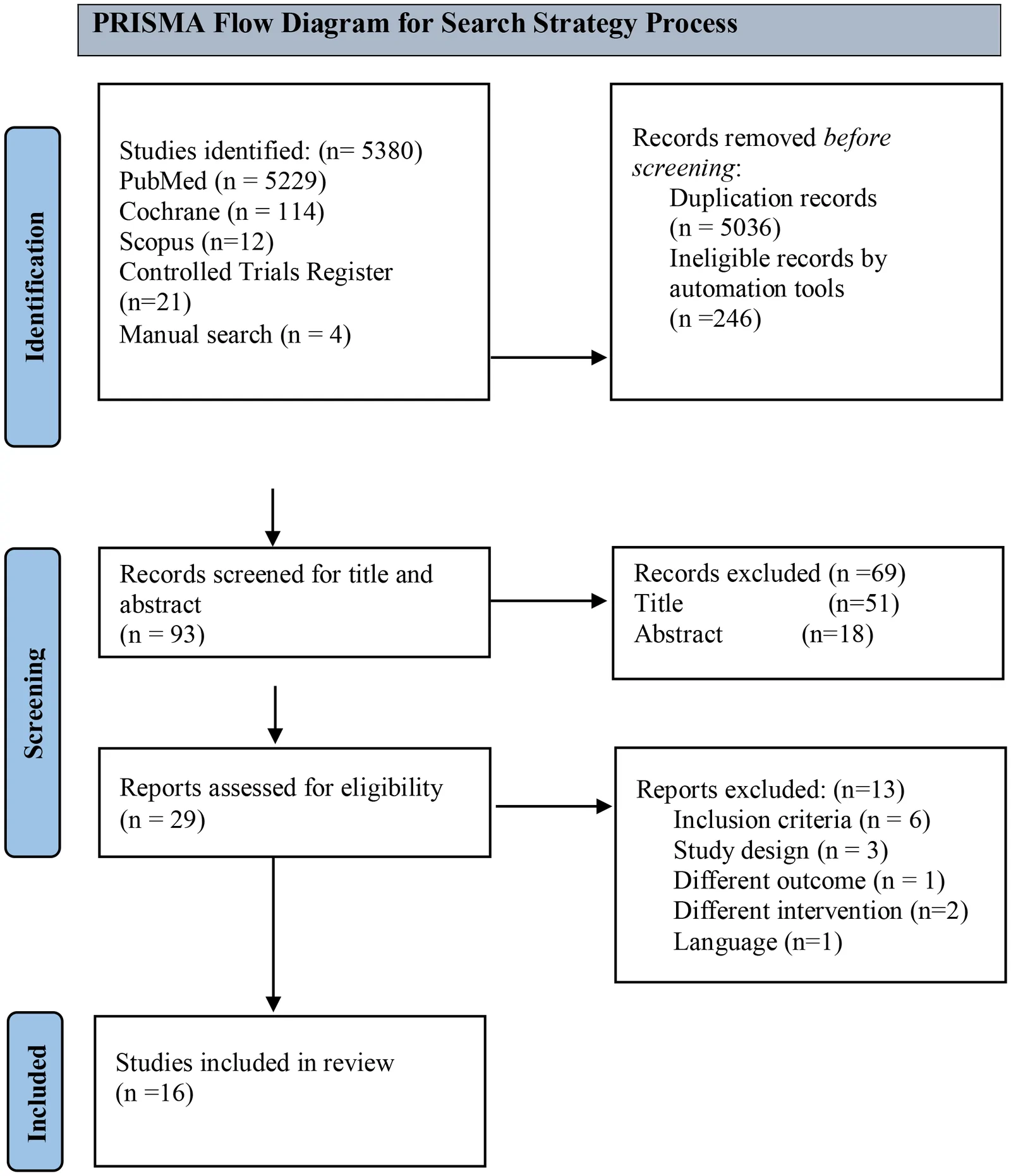
PRISMA flow diagram for search strategy process.

After initially excluding publications based on the title or abstract, 29 full-text articles were extracted for a more comprehensive evaluation. Following full-text screening, another 13 of these research (26–38) were excluded due to reasons such as lack of a direct comparison between early and delayed insulin initiation, restricted access, incomplete outcome reporting, or high risk of bias in observational data (**Supplementary Table 3**). A total of 16 relevant articles were included in this systematic review. The sequence of identification, and screening process was displayed in PRISMA Flow Diagram (**Figure 1**).

### 3.2 Characteristics of the included studies

A total of 16 articles, comprising 44, 045 participants, met the eligibility criteria for this systematic review (**Table 1**). The participant age range was from 44.8 ± 9.7 years to 64.9 ± 13.5 years. While most studies focused on middle-aged and older adults, a few studies included younger individuals (<40 years old). However, none specifically stratified outcomes for this subgroup. Some observational studies suggested that younger patients had a better response to early insulin initiation, with greater HbA1c reductions and less weight gain, but further subgroup analysis was not consistently reported across studies.

**Table 1:** Characteristics of the included studies.

Author/country	Study design	Sample size	Duration of follow up/years	Characteristics of the participants				Intervention & comparator	Outcome
				Age/year	Gender [n] males/females	BMI/kg/m²	Onset of diagnosis		
(Chon et al., 2010) Korea	Retrospective cohort study	61	4 years	48.3 ± 11.5	37/24	25.0 ± 3.5	At the time of diagnosis	Early insulin therapy with biphasic or prandial regimens; single-arm within-cohort evaluation targeting HbA1c <7% (no external comparator)	β-cell function and glycemic control to achieve a (HbA1c) level of less than 7%.
(Xie L et al., 2011) USA	Retrospective cohort analysis	1682	1 year	55.00 ± 10.1	729/953	25.22 – 3.64	1 year	Basal insulin via disposable pens: Gla-100 (glargine) vs Det (detemir); comparative real-world cohort.	Glycemic control, measured by A1C level Adverse events
(Hu Y et al., 2011) China	RCT	48	1 year	50.6 ± 7.9	34/14	25.7 ± 3.3	Newly diagnosed	CSII vs MDI in newly diagnosed T2DM.	Glycemic control therapy; sensitivity and β-cell function
(Harrison et al., 2012) USA	RCT	58	3.5 years	44.8± 9.7	37/21	35.6 ± 6.6	2 months	Insulin-based intensive therapy vs TOT (triple oral therapy).	β-cell function and Glycemic control to achieve a (HbA1c) level of less than 7%.
(Gerstein et al., 2012) International multicenter	RCT	12, 537	6.2 years	63.6 ± 7.8	8149/4388	29.8 ± 5.2	5.5 ± 6.1	Insulin glargine (basal) vs standard care (non-insulin therapy per guideline-based routine care).	Glycemic control Cardiovascular adverse events
(Zeng et al., 2012) China	RCT	59	2 weeks	50.37 – 9.82	30/29	25.46 – 3.12	Newly diagnosed	Basal insulin monotherapy vs CSII during a 2-week induction phase.	Glycemic control therapy; sensitivity and β-cell function
(Pistrosch et al., 2013) Germany	RCT	75	36-week	60.7 ± 9.2	44/31	29.2 ± 4.6	Newly diagnosed 2.8 ± 1.4	Insulin glargine (basal) vs metformin (early T2DM).	glucose control, microvascular function and preserve insulin secretion
(Levin et al., 2016) USA	Retrospective database analysis	1830	1 year	56.7 ± 13.9	976/854	34.7± 8.9	5 years	Basal insulin initiation with timing-stratified cohorts; outcomes compared between earlier vs later initiators of basal insulin among patients previously on OADs.	Glycemic control Body weight change Health care cost
(Nyström et al., 2017) Sweden	Observational registry study	15, 466	3.84years	64.9 ± 13.5	4838/10628	–	3.4 years	Second-line insulin (after metformin) vs DPP-4 inhibitor (after metformin).	all-cause mortality, fatal and nonfatal cardiovascular disease (CVD), and severe hypoglycemia
(Wang et al., 2019) China	RCT clamp-based study	124	2years	47.33 ± 9.45	92/32	25.0 ± 3.25	Newly diagnosed 0.30 ± 1.03	Short-term IIT/CSII protocol in newly diagnosed T2DM; single-arm assessment with clamp-based outcomes (no external comparator).	Glycemic control therapy; sensitivity and β-cell function
(Bailey et al., 2019) USA	Retrospective cohort study	3012	0.5 year	60.2 ± 12.3	1603/1403	33.6 ± 7.0	12-months	Gla-300 vs Gla-100 among insulin-naïve patients (real-world DELIVER-Naïve).	Glycemic control Hypoglycemia
(Karacaer et al., 2021) Turkey	Retrospective study	65	1 year	48.03 ± 10.78	41/24	30.4 ± 5.8	1.21 ± 0.53 years	Short-term IIT in newly diagnosed T2DM; single-arm pre–post evaluation (no external comparator).	Long-term glycemic control
(Retnakaran et al., 2021) Canada	RCT	108	2 years	56 ± 9	56/52	33.7 ± 7.2	(median 1.3 years’ duration e 0.5-3.0 years)	MET + short-term IIT (induction/maintenance) vs MET alone (maintenance), evaluating β-cell function (ISSI-2) and HbA1c.	Glycemic control therapy; sensitivity and β-cell function
(Pigeyre et al., 2022) Sweden	RCT	7017	6.2 years	≥50 Median age; 63 [58–69] years)	4616/2401	Median 29.51 (26.51–33.02)	5.16 ± 5.74 years	Insulin glargine (basal) vs standard care; subgroup validation of T2DM clusters for complications risk.	Risk of diabetes‐related microvascular and macrovascular complications and all‐cause mortality Glycemic control
(Jeon et al., 2022) Korea	Retrospective cohort study	1, 804	5 years	52.8± 12.21	757/1047	25.22 ± 3.64	1 year after diagnosis	Insulin regimens (premixed, basal, short-acting) vs OAD treatment in newly diagnosed T2DM.	Risk of diabetes‐related microvascular and macrovascular complications and all‐cause mortality
(Retnakaran et al., 2023) Canada	RCT	99	2years	61.2 + 18.0	60/33	33.4 + 6.7	1.9 + 1.5	Short-term IIT with 2-year follow-up on β-cell stabilization; single-arm cohort with determinants (no external comparator).	Glycemic control therapy; sensitivity and β-cell function

Body mass index; EIT, Early insulin therapy; HbA1C, Glycosylated hemoglobin; Gla, Insulin glargine; DET, insulin detemir; RCT, randomized controlled trial; CSII, Continuous subcutaneous insulin infusion; MDI, multiple daily insulin injections; TOT, Triple oral therapy; OAD, oral anti-diabetics drugs; IIT, intensive insulin therapy.

In terms of study design, nine studies were randomized controlled trials (RCTs) (3, 7, 39–45). The remaining studies included six retrospective observational studies (46–51), one observational registry (52). The pooled sample sizes for the RCTs and observational studies enrolled were 20, 125 and 23, 920 patients respectively.

Diabetes mellitus (DM) was diagnosed in all studies according to WHO criteria. The included studies had a diverse range of body mass indices (BMI), with mean BMI values ranging from 25.0 ± 3.5 to 35.6 ± 6.6 kg/m². Early insulin initiation (within 5 years of diagnosis) was reported in most studies, while others examined insulin use in patients with established disease. The duration of follow-up across included studies ranged from 2 weeks (45) to 6.2 years (44). Short-term ‘induction-phase’ studies assessed immediate glycemic and β-cell responses and therefore cannot inform long-term durability or complication risks.

Across studies, populations differed in disease duration, baseline glycemic status, and prior use of oral agents. Overall, **Table 2** highlights the wide variation in study designs and patient characteristics, underscoring the diversity of evidence informing early insulin initiation in T2DM.

**Table 2:** Certainty of evidence for key outcomes from randomized controlled trials (GRADE).

Outcome	No. of studies	Study design	Risk of bias (over all)	Inconsistency	Indirectness	Imprecision	Publication bias	Overall certainty of evidence
Glycemic control								
Reduction of HbA1c	8 RCTs (3, 39-45)	High	Not serious	Not serious	Not serious	Not serious	Undetected	High
β-Cell Function Preservation	6 RCTs (3, 7, 40, 42, 43, 45)	High	Not serious	Serious	Not serious	Not serious	Undetected	Moderate
Safety profile & adverse events								
Hypoglycemia (Overall Incidence)	6RCTs (3, 7, 39, 41, 44, 45)	High	Serious	Not serious	Not serious	Not serious	Undetected	Moderate
Weight Gain	5 RCTs (3, 7, 41, 43, 44)	High	Serious	Serious	Not serious	Serious	Undetected	Low
Cardiovascular Outcomes (MACE)	2 RCTs (39, 44)	High	Not serious	Not serious	Serious	Serious	Undetected	Low
Microvascular Complications	1 RCT (1)	High	Not serious	Not assessable	Serious	Serious	Undetected	Low

### 3.3 Characteristics of interventions and outcomes

The characteristics of the interventions and outcomes are presented in (**Table 3**). The studies included in this review evaluated a range of insulin regimens—including basal insulin (glargine U100/U300, detemir), premixed formulations, prandial insulin, and intensive strategies such as MDI or CSII. Regardless of the insulin type, all interventions targeted improved glycemic control and were compared either with alternative insulin regimens or with oral antidiabetic medications.

**Table 3:** Characteristics of the intervention and the outcomes.

Author	Glycemic control				BMI/kg/m²		Safety profile & adverse events	Conclusion
	Assessment of B function	HbA1C/%						
		Baseline		After	Baseline	After		
(Chon et al., 2010)	Serum insulin and C-peptide levels HOMAIR IGI, ACR HOMAβ	10.7 ± 1.8%		6.2 ± 1.1%	24.7 ± 3.5	25.2 ± 3.5	Increase body weight during the study period	EIT significantly improves β-cell function and facilitates long-term glycemic control
(Xie L et al., 2011)	–	9.4 ± 2.0		8.1 ± 1.6	25.22 – 3.64	–	The mean number of hypoglycemic events per patient per year (0.23 ± 1.26) No association between early insulin use and the risk of macrovascular complications and all-cause mortality increased microvascular complication	Mean change in A1C from baseline levels of −1.23 (95% CI, −1.39%, −1.06%)
(Hu Y et al., 2011)	AIRins during IVGTT- HOMA-IR β -cell function index	10.0 ± 2.2		6.5 ± 0.7	25.7 ± 3.3	37.4 25.2 ± 3.3	–	- A remission rate of ~44% and improved β -cell function, as measured by HOMA-b and AIRi.
(Harrison et al., 2012)	AUCC Total insulin secretion The ratio of AUCC to AUCG	6.0 ± 0.5		6.4 ± 0.8	35.6 ± 6.6	37.5 ± 9.3	- Weight increased from 102.2 ± 24.9 to 106.2 ± 31.7 kg - Mild hypoglycemia - Two participants, three events of severe hypoglycemia	- 79% met ADA guidelines of HbA1c, 7% - β-cell function preserved for at least 3.5 years after diagnosis of type 2 diabetes
(Gerstein et al., 2012)	–	5.8–7.2		5.8–6.8	29.8 ± 5.2	–	Associated with a modest increase in hypoglycemic episodes and in weight (a median of 1.6 kg)	Neutral effect on cardiovascular outcomes and cancers Maintained near-normal glycemic control and slowed progression of dysglycemia,
(Zeng et al., 2012)	homeostasis model (HOMA-B and HOMA-IR	10.85 ± 2.40		Basal 9.91 ± .0.95 CSI1; 10.03 ± 1.91	25.46 – 3.12	–	Minor hypoglycemic episodes or classical symptoms of hypoglycemia was observed in both groups [31.25% in CSII vs. 22.22% in the basal insulin group, P < 0.05] No obvious weight gain was observed in either group	88.14% of patients achieved fasting glycemic control target, HbA1c decreased nearly 1% b-cell function, especially AIR, was greatly improved, HOMA-B and HOMA-IR were also improved
(Pistrosch et al., 2013)	(HOMA B) and insulin resistance (HOMA IR). HOMA	7.2 ± 0.7		6.36 ± 0.4	29.2 ± 4.6		patients gained weight, significantly with increase in waist circumference of 1.1 ± 3.7 cm Hypoglycemia—occurred rarely	Pronounced reduction in mean IG and AUC with insulin glargine, HOMA B Beta-cell function were significantly more improved
(Levin et al., 2016)		9.3 ± 2.3		< 7	34.7 ± 8.9		3% of patients had experienced ≥1 hypoglycemic event	Better clinical outcomes with insulin therapy, with no increase in overall costs
(Nyström et al., 2017)	–	–		–	–	–	Severe hypoglycemia	Initiation of insulin, compared with DPP-4i treatment, was associated with an increased risk of subsequent all-cause mortality, fatal and nonfatal CVD.
(Wang et al., 2019)	Insulin AUC/glucose AUC	11.07 ± 2.05		11.02 ± 2.40	25.0 ± 3.25	25.12 ± 3.26	The baseline BMI was maintained throughout the study	First-phase insulin secretion was restored, and the GIR was significantly improved (P < 0.0001) Glycemic remission rates were 47.6% and 30.7% after 12 and 24 months of follow-up, respectively.
(Bailey et al., 2019)		9.59 ± 1.96		8.07 ± 2.08	33.6 ± 7.0	–	Hypoglycemia during the 6-month baseline period (5.6% and 5.3%, in both group	During 90–180‐day follow‐up, mean HbA1c decreased significantly (P < 0.001)
(Karacaer et al., 2021)	–	11.25 ± 1.96		6.67 ± 1.0	30.4 ± 5.8	–	–	The target HbA1c value after SIIT was achieved by 67.3% and 71.0% of participants after three months and 12 months, respectively.
(Retnakaran et al., 2021)	Insulin Secretion Sensitivity Index-2 (ISSI-2) at an oral glucose tolerance test	6.6% ± 0.6%		6.4 ± 0.1	33.7 ± 7.2	32.7 ± 0.3	no episodes of severe hypoglycemia The absolute rates of hypoglycemia per patient-week on IIT were modest, with rates of 0.12 and 0.08	ISSI-2 at 2 years was not improved by (MET + IIT) HbA1c at 2 years did not differ between MET and MET + IIT (6.3% ± 0.1% vs. 6.4% ± 0.1%, P = .46), Relative stabilization of beta-cell function
(Pigeyre et al., 2022)	–	Median 6.50 (6.95–7.23)			–	Median 29.51 (26.51–33.02)	increase in the incidence of impaired kidney function (49) Macroalbuminuria [56%] and retinopathy & coronary event	Significantly decreased hyperglycemia by 19% Clustering for the management of diabetes would improve patient risk stratification for diabetes- related consequences
(Jeon et al., 2022)	–	FBG; 224.54 ± 97.8			25.22 – 3.64	–	–	No association with the risk of macrovascular complications and all‐cause mortality compared with OAD treatment; The risk of microvascular complications was higher in the insulin group.
(Retnakaran et al., 2023)	ISSI-2 Insulinogenic index/HOMA-IR Matsuda index	6.7 + 0.7	6.3 + 0.6		33.4 + 6.7	32.3 + 6.9	change in BMI during the maintenance phase may have emerged as an additional significant negative predictor	55 of 99 patients with T2DM achieved sustained stabilization of beta-cell function over 2-years

HOMA-IR the homeostasis model assessment of insulin resistance; IGI, the insulinogenic index; ACR, the acute C-peptide response; HOMA β, l the homeostasis model assessment of β-cell function; AIRins, Acute insulin response of insulin; ISSI-2, Insulin Secretion-Sensitivity Index-2.

Glycemic outcome was primarily assessed using blood glucose levels or glycated hemoglobin (HbA1c). All studies except for Nyström et al. (2017) (52) defined their primary outcomes in terms of glycemic control, with HbA1c levels aimed at achieving <7%. Other methods for measuring glycemic control included β-cell function tests and various metrics such as the area under the curve (AUC) for glucose and C-peptide. Some studies also examined insulin resistance through HOMA-IR or fasting blood glucose levels (3, 38, 46), while hypoglycemia was defined as blood glucose ≤3.9 mmol/L, with adverse events documented in multiple studies (3, 35–38, 48–50, 52). Weight gain (>2 kg) was observed in some studies, measured using body weight assessments at baseline and follow-up (ranging from 6 months to 2 years) (3, 38, 46, 48, 50). While this weight gain was statistically significant in some studies, it was generally moderate and did not lead to substantial discontinuation of insulin therapy. The clinical implications remain debated, as some studies suggested that early insulin initiation contributes to better glycemic control, outweighing the risks associated with modest weight gain. Long-term outcomes, including microvascular and macrovascular complications, were evaluated in studies with extended follow-up periods.

### 3.4 Risk of bias

All included studies were assessed for methodological quality and risk of bias (RoB) using the Cochrane Collaboration tool (23). This evaluation considered selection bias (allocation concealment and random sequence generation), performance and detection bias, attrition and reporting bias, as well as any other biases not previously mentioned. JBI checklist was used to evaluate the risk of bias for cohort studies (**Supplementary Tables 4**, **5**). For RCT (3, 7, 39, 40, 43–45);, the studies reduced selection bias by concealing the allocation sequences from all included participant, and using random tool that generates the random sequence, although random sequence generation is not clear at Hu Y et al., 2011 (40) and Pistrosch et al., 2013 (41). Wang et al., 2019 (42) conducted a single arm interventional trial so it lacks randomization and consequently allocation concealment. Blinding was limited across several trials due to the nature of insulin interventions, which may introduce performance bias. In Harrison et al. (2012), a total of 13 out of 58 participants withdrew from the study across both groups. No evidence of reporting bias or other sources of bias was identified in the included trials.

In all observational studies (46–52) the groups of participants similar and recruited from the same population, and the exposure and outcomes measured in a valid and reliable way. In two studies confounders were not identified (48, 52). Levin et al., 2016 and Xie L et al., 2011 stated strategies to deal with confounding factors.

Strategies to address incomplete follow up were not utilized across all studies. RoB judgments informed our GRADE certainty ratings, with domains such as allocation concealment/blinding (RCTs) and confounding/incomplete follow-up (observational studies) considered when rating risk of bias. Where RoB, inconsistency, or imprecision were present, we downgraded the certainty of evidence for the affected outcomes.

**Figure 2:**
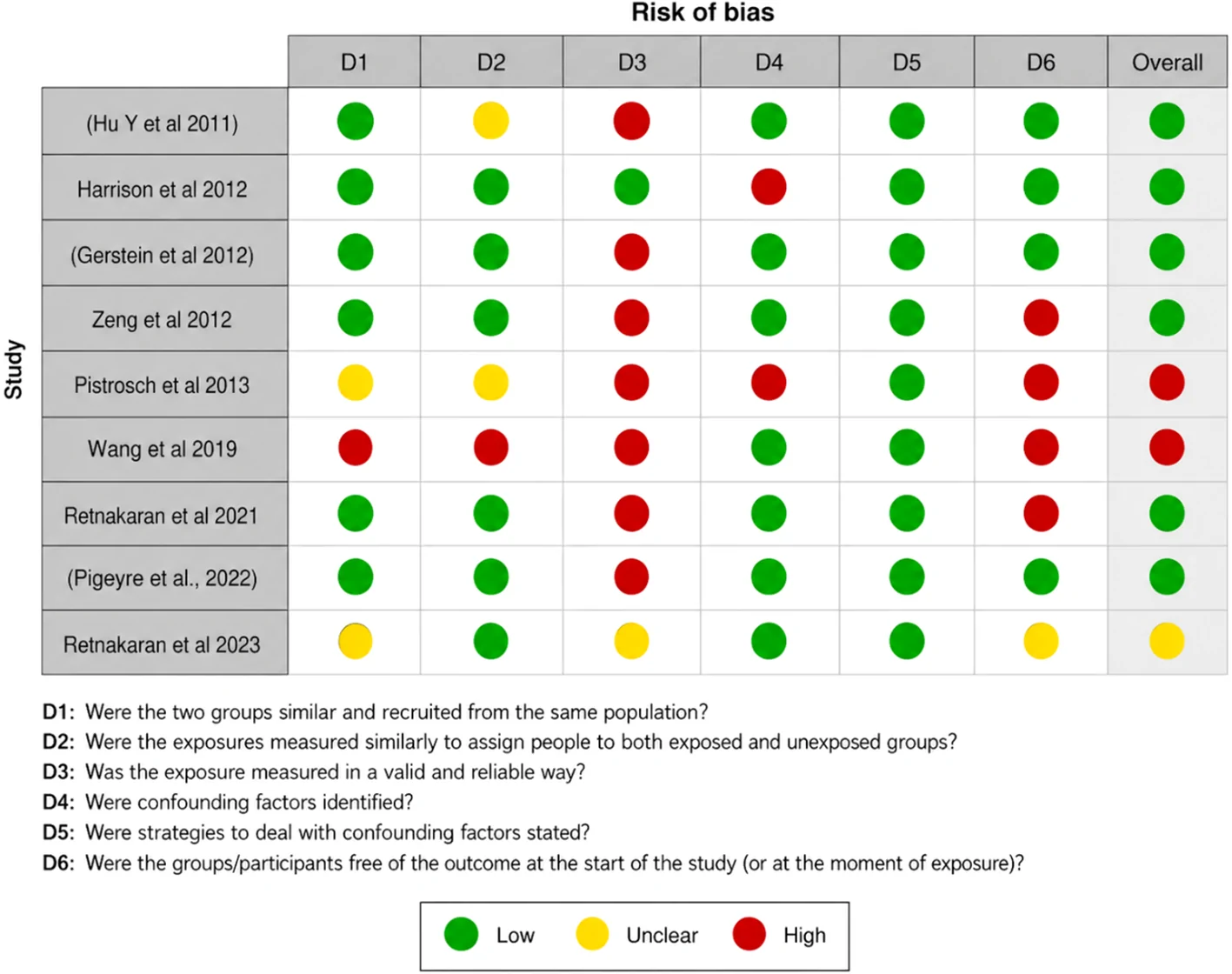
Risk-of-bias assessment of randomized controlled trials (RoB 2). Traffic-light plot showing study-level judgments across domains. Green = low risk, yellow = some concerns, red = high risk.

**Figure 3:**
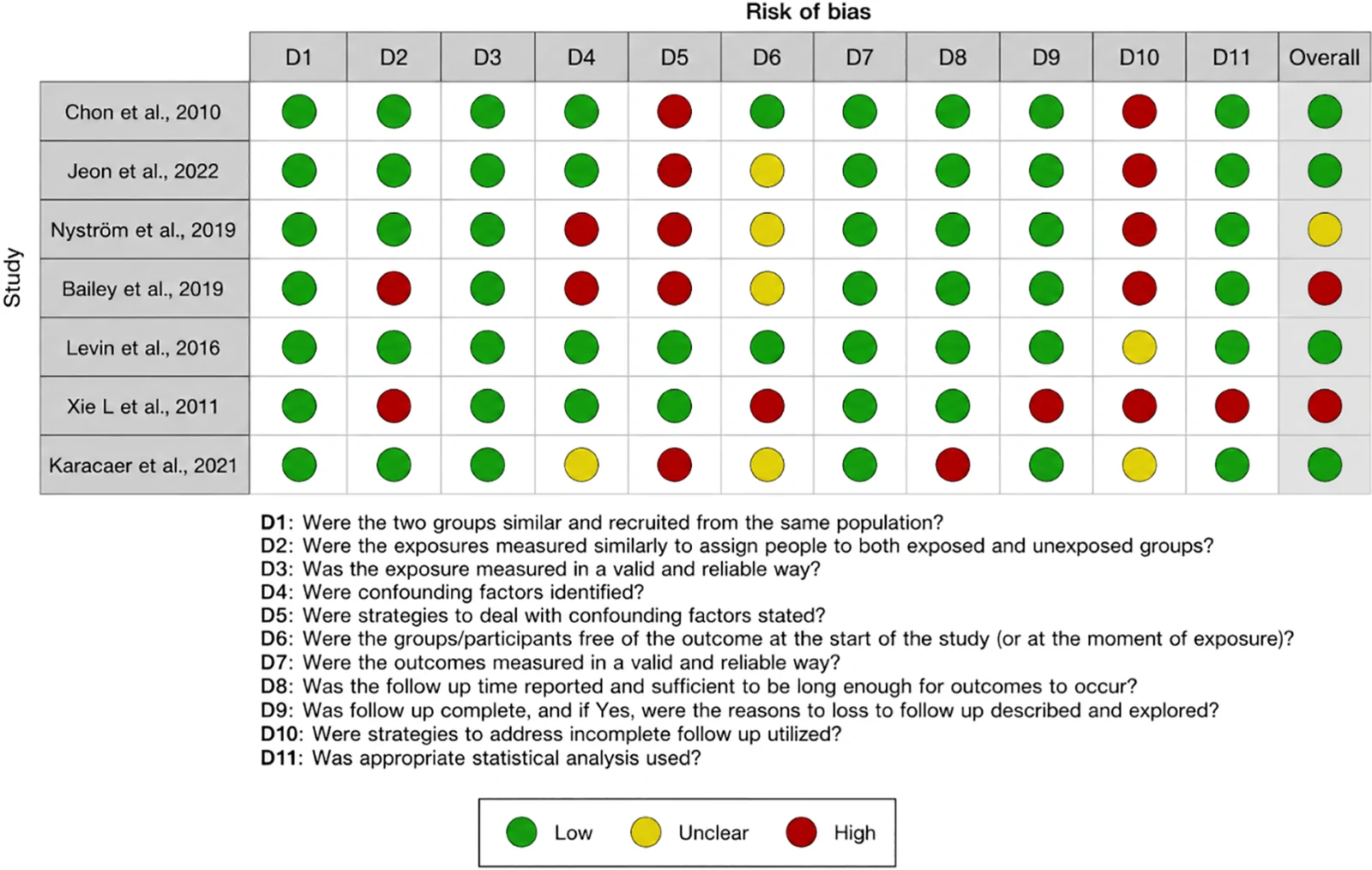
Risk-of-bias assessment of cohort studies (JBI). Traffic-light plot showing study-level judgments across domains. Green = low risk, yellow = unclear, red = high risk.

The results of the RoB assessments are presented in **Figure 2** for RCTs and **Figure 3** for observational studies, with corresponding summary plots provided in **Supplementary Figures 2** and **S3** (53). Across RCTs, the most frequent concerns were limited blinding and small sample sizes for long-term outcomes. In observational studies, residual confounding and incomplete follow-up were the most prominent sources of bias. These patterns were reflected in the GRADE assessments, with higher certainty observed for short-term glycemic outcomes and lower certainty for long-term cardiovascular, microvascular, and safety outcomes.

### 3.5 Quality (certainty) of evidence

To strengthen the methodological rigor, the Grading of Recommendations Assessment, Development and Evaluation (GRADE) approach was used to assess the certainty of evidence across key outcomes. The certainty of evidence was evaluated based on study design, risk of bias, inconsistency, indirectness, imprecision, and publication bias. Evidence from randomized controlled trials (RCTs) was initially rated as high certainty, while observational studies were initially rated as low certainty, with subsequent downgrading or upgrading applied as appropriate.

The overall certainty of evidence was categorized into four levels:

- High certainty: We are very confident that the true effect lies close to that of the estimate.
- Moderate certainty: We are moderately confident in the effect estimate; the true effect is likely close to the estimate, but there is a possibility that it is substantially different.
- Low certainty: Our confidence in the effect estimate is limited; the true effect may be substantially different from the estimate.
- Very low certainty: We have very little confidence in the effect estimate; the true effect is likely to be substantially different from the estimate.

The detailed GRADE assessments for RCTs and observational studies are presented in **Tables 2**, **4**, respectively.

**Table 4:** Certainty of evidence for key outcomes from observational studies (GRADE).

Outcome	No. of studies	Study design	Risk of bias (overall)	Inconsistency	Indirectness	Imprecision	Publication bias	Overall certainty of evidence
Glycemic control								
Reduction of HbA1c	5 Observational Studies (46, 48-51)	Low	Serious	Not serious	Not serious	Not serious	Suspected	Very low
Safety profile & adverse events								
Hypoglycemia (Overall Incidence)	4 Observational Studies (46, 48-50)	Low	Not serious	Serious	Not serious	serious	Suspected	Very low
Weight Gain	3 Observational Studies (46, 49, 50)	Low	Very serious	Serious	Not serious	Very serious	Undetected	Very low
Cardiovascular Outcomes (MACE)	2 Observational Studies (47, 52)	Low	Very serious	serious	Serious	Serious	Undetected	Very low
Microvascular Complications	2 Observational Studies (47, 50)	Low	Very serious	Not serious	Moderate	Serious	Undetected	Very low
Healthcare Costs & Utilization	1 Observational Study (49)	Low	Very serious	Not assessable	Serious	Very serious	Suspected	Very low

## 4 Discussion

### 4.1 Summary of main findings

This systematic review identified 16 studies, totaling 44, 045 individuals, published between 2010 and 2024, that investigated the impact of early insulin treatment beginning in type 2 diabetic mellitus (T2DM). Overall, most studies found that starting insulin within five years after diagnosis considerably improved glycemic control, as judged by HbA1c levels below 7%. The term “glycemic control” was assessed in various studies using multiple metrics, including Hemoglobin A1c (HbA1c) levels, fasting blood glucose (FBG), and postprandial glucose (PPG). However, there was variability in the measurement methods across studies. While some studies relied solely on HbA1c as a long-term indicator of glycemic control, others incorporated self-monitored blood glucose (SMBG) levels or continuous glucose monitoring (CGM) data. This variability necessitated a careful synthesis of the findings, accounting for differences in measurement consistency. Studies that reported HbA1c provided a more standardized comparison, while those using alternative methods required additional interpretation regarding the timeframe and reliability of glycemic monitoring. Future studies should adopt uniform measurement criteria to facilitate direct comparisons and meta-analyses. The certainty of evidence was highest for short-term glycemic outcomes, whereas long-term cardiovascular and safety outcomes were limited by imprecision, small sample sizes, and, in many cases, reliance on observational data.

Early insulin treatment was associated with preservation or stabilization of β-cell function in several studies (3, 40, 46). However, adverse events such as hypoglycemia, possible weight gain, and microvascular problems were reported, emphasizing the significance of close patient monitoring. Furthermore, one research found a probable link between early insulin treatment and renal problems, emphasizing that potential dangers must be evaluated against benefits.

### 4.2 Comparison with previous studies

Our results are consistent with previous research indicating that early insulin introduction may provide superior long-term glycemic control (6, 54). For example, Sidorenkov et al. (2018) (6) found that starting insulin treatment during the first few years after T2DM diagnosis led to decreased HbA1c levels throughout a three-year follow-up period. Our evaluation, which includes more recent research (2018-2022), supports previous findings of possible β-cell preservation and better glycemic outcomes. Previous research has often focused on insulin as a late-stage treatment, utilized after oral hypoglycemic medications fail to maintain appropriate glucose control (55, 56). However, delaying the initiation of insulin may allow progressive β-cell decline, leading to worsening hyperglycemia and ultimately necessitating intensification of therapy to maintain glycemic control (57). Current ADA/EASD guidelines emphasize individualized, patient-centered approaches and recommend insulin initiation particularly in cases of severe hyperglycemia, symptomatic disease, or evidence of catabolism (58). This intensification often involves insulin treatment, with 20% to 30% of adults with type 2 diabetes reported to use insulin at any given time (56).

Preserving residual β-cell activity, reported in this narrative review, may lead to long-term metabolic benefits, particularly in individuals with shorter illness durations (59).

Hassan et al. (2022) found a significantly higher rate of adverse cardiovascular outcomes in insulin-treated diabetes mellitus patients following percutaneous intervention (PCI) (60). Other studies have reported an increased risk of cardiovascular events in patients receiving early insulin therapy, particularly in those with pre-existing atherosclerotic disease or higher baseline cardiovascular risk (61–63). These findings suggest that heightened insulin exposure may contribute to weight gain, hyperinsulinemia, and pro-inflammatory pathways that could adversely affect the cardiovascular system over time. A number of the trials we looked at had either no effect or a slightly negative effect on major cardiovascular complications. Hazard Ratios of incident cardiovascular (CV) events were assessed in two RCT studies included in this review (39, 41, 44). No differences were observed in the incidence of cardiovascular diseases. One retrospective observational study reported a significant higher risk of overall microvascular complications for insulin group but no increased risks of overall macrovascular complications (47). He suggested tight glycemic control achieved through high-dose insulin therapy may contribute to microvascular complications. Nyström et al. measured the adjusted HRs and observed that initiating insulin as a second-line therapy after metformin monotherapy in patients with type 2 diabetes was associated with a higher risk of all-cause mortality and cardiovascular events compared to initiating dipeptidyl peptidase-4 (DPP-4) inhibitors (52). He suggested this higher risk may be due to advanced or poorly controlled diabetes when insulin therapy is initiated. Chronic hyperglycemia induces endothelial dysfunction and oxidative stress, leading to accelerated atherosclerosis and increased cardiovascular risks (9). Furthermore, insulin therapy is linked to a higher risk of hypoglycemic episodes and weight gain, both of which can exacerbate insulin resistance and negatively impact cardiovascular health. Notably, the remaining studies lacked direct cardiovascular event reporting and focused on β-cell function, total health care costs and glycemic control, which limited the ability to assess the full scope of risk (3, 7, 40, 42, 43, 45, 46, 48–51). This inconsistency highlights the need for future studies to incorporate comprehensive cardiovascular monitoring and direct outcome reporting to improve risk assessment. Furthermore, the findings underscore the need for a targeted approach to manage glycemic variability, rather than focusing solely on HbA1c reduction, to mitigate cardiovascular risks in diabetic patients. This supports the idea that the overall cardiovascular benefits of starting insulin therapy early may depend on the patient, their BMI, and any other conditions they may have, as well as the type of insulin they are taking and how long they are being followed up on. Taken together, this emerging body of evidence underscores the complexity of early insulin initiation and highlights the need for careful patient selection and monitoring, especially in those with a high cardiovascular risk profile. In contrast, Luo et al. (2024) observed no significant difference in the risk of CHD, but reported a 31% lower risk of incident stroke and a 28% lower risk of hospitalization for HF among newly diagnosed T2D patients who received early insulin therapy compared with those who did not receive such treatment (64).

### 4.3 Adverse events and barriers to early insulin initiation

Hypoglycemia and weight gain emerged as the two most frequently reported challenges associated with early insulin initiation in this review. Similarly, Harrison et al. (2012) identified treatment failure, particularly among obese patients, as a key barrier to implementing insulin therapy. This conclusion is comparable with the findings of Heller et al. (2020), who found a 25% of people with T2D taking insulin for >5 years were found to have severe hypoglycemic events (65). Furthermore, weight gain associated with insulin therapy is exacerbated by the presence of multiple co-morbidities, including obesity, making the management of their diabetes and obesity challenging (66). Home et al. (2015) reported that weight gain at 4 years, following of insulin initiation, increased for the entire population by 2.5 kg, with most of the gain occurring in the first year (67).

Older adults and individuals with renal insufficiency tend to be more prone to these adverse effects. Freeman, 2019 (68) found that patients over 65 had a 1.5-fold higher risk of severe hypoglycemia, which they attributed to reduced physiological reserves, polypharmacy, and comorbidities. Our results confirm these observations, since almost half of the included studies found that baseline renal function increased hypoglycemia risk, emphasizing the need of personalized insulin titration and attentive monitoring in these groups.

Most large trials of intensive glycemic control confirmed that intensive glycemic control significantly decreased rates of microvascular complications in patients with short-duration type 2 diabetes, compared with those managed by oral hypoglycemic agents alone (58, 69). Conversely, several of the studies in our evaluation revealed an increased risk of microvascular problems (e.g., retinopathy, nephropathy) with early insulin administration. Their analysis suggested that the interaction of residual insulin resistance, underlying inflammation, and early hyperinsulinemia may exacerbate microvascular damage in some individuals. Although the specific processes are unknown, these results highlight the multifaceted nature of T2DM therapy, with glycemic control being just one aspect of care.

Apart from clinical hazards, various emotional and logistical impediments might stymie the early acceptance of insulin treatment (70, 71). Arshad et al. (2019) (72) highlighted dread of injections, a lack of patient education, and misunderstandings about insulin that contribute to “end-stage” illness as major barriers. Our research also discovered deficient patient adherence in a subgroup of observational studies, with authors attributing low adherence rates to needle fear and insufficient insulin delivery technique training. These hurdles are consistent with prior research by Kalra et al. (2020), who shown that organized diabetic education programs might significantly improve patient acceptability of early insulin treatment (73).

### 4.4 Recent studies and innovations

Insulin analogs and continuous glucose monitoring (CGM) technologies have been investigated in a number of recent clinical studies may reduce the risk of common side effects such as hypoglycemia (74, 75). The early findings of these studies highlight the changing landscape of insulin treatment, despite the fact that we did not include them in our analysis owing to eligibility requirements or publication timelines. The incorporation of such advanced therapies has the potential to further optimize the risk–benefit balance of early insulin introduction by lowering the risk of hypoglycemia and making insulin titration more straightforward.

### 4.5 Strengths and limitations in context

This review has several strengths. It addresses an important and timely clinical question in type 2 diabetes management, employs a comprehensive and systematic search strategy across multiple major databases, and follows PRISMA guidelines. Clear inclusion and exclusion criteria were applied, and both randomized controlled trials and observational studies were considered, providing a broad overview of the available evidence. Risk of bias was formally assessed, using validated risk-of-bias assessment tools.

Nonetheless, several limitations warrant consideration. First, there is no universally accepted guideline definition of “early” insulin initiation. While clinical guidelines (ADA/EASD, AACE) recommend insulin initiation in cases of severe hyperglycemia or catabolic states, most clinical trials and systematic reviews—including our study—have operationalized “early” as initiation within five years of diagnosis. This pragmatic definition facilitates comparability across studies but may not fully reflect guideline-driven contexts. Second, substantial clinical and methodological heterogeneity across studies, including differences in patient characteristics, insulin regimens, and outcome definitions, limits direct comparability and affects generalizability. Although this diversity reflects real-world practice, it complicates interpretation, particularly for specific subgroups such as older adults and individuals with multiple comorbidities. Third, the influence of confounding factors such as age, disease duration, obesity, and comorbidities was not consistently adjusted across studies, which may have affected observed outcomes. The variability in follow-up duration, with some studies reporting short-term outcomes, limits conclusions regarding long-term efficacy and safety. Fourth, the potential misclassification of LADA as type 2 diabetes cannot be excluded, since autoimmune antibody testing was not consistently reported; such cases may bias the results toward greater insulin responsiveness. Fifth, β-cell function outcomes were assessed using heterogeneous surrogate measures (e.g., HOMA-β, insulinogenic index, C-peptide, ISSI-2), which are not standardized across studies and have methodological limitations; these results should therefore be interpreted with caution. Sixth, while we performed a structured risk-of-bias assessment, the overall certainty of evidence varied. Our conclusions reflect this: short-term glycemic benefits (HbA1c reduction) are supported primarily by RCTs with generally lower RoB, yielding moderate certainty, whereas long-term cardiovascular and microvascular outcomes and safety endpoints rely heavily on observational data with residual confounding and incomplete follow-up, leading to downgraded certainty. Variability in outcome definitions and small event counts further contributed to imprecision, reinforcing cautious interpretation of long-term safety. Finally, as with any systematic review, publication bias and incomplete reporting across studies may have influenced our synthesis.

Future research should therefore prioritize longer follow-up durations, standardized β-cell assessment methods, rigorous exclusion of autoimmune diabetes, and subgroup analyses and report standardized strata (age bands, duration categories, comorbidity indices, and concomitant therapies) to enable robust pooled estimates. Studies evaluating patient-centered outcomes, including cost-effectiveness, satisfaction, and quality of life, as well as strategies to overcome barriers to early insulin initiation, are particularly needed to inform practice and policy.

### 4.6 Clinical implications

All available evidence, including findings from this review and prior studies, indicates that early insulin initiation can effectively improve glycemic control in type 2 diabetes mellitus and may contribute to preservation of β-cell function. In clinical practice, early insulin should be considered in patients presenting with marked hyperglycemia (HbA1c ≥9–10%), symptomatic hyperglycemia, or catabolic features such as weight loss or ketosis. It may also be appropriate in individuals with rapid deterioration of glycemic control despite combination therapy or in those with contraindications to other glucose-lowering agents.

Patient selection remains critical. Younger individuals, those with shorter disease duration, and those without significant comorbidities are more likely to achieve substantial glycemic benefit with a lower risk of complications. In contrast, older adults and patients with multiple comorbidities, chronic kidney disease, or prior hypoglycemia require careful risk–benefit assessment, as they are more susceptible to adverse events, including hypoglycemia and weight gain.

From a practical perspective, early insulin initiation is typically best implemented using a basal insulin regimen, with continuation of metformin and cardioprotective agents when appropriate, followed by gradual titration to achieve glycemic targets. The use of newer insulin analogs, continuous glucose monitoring, and structured patient education can further improve safety and adherence. Overall, early insulin therapy should be integrated within a personalized treatment strategy, balancing glycemic benefits with patient-specific risks and preferences.

### 4.7 Future directions

Building on the gaps identified in this and other reviews, future research should focus on developing standardized definitions of “early” insulin initiation, harmonized outcome measures, and robust long-term evaluations of cardiovascular and microvascular outcomes. Studies examining cost-effectiveness, quality of life, and patient-centered strategies—including how to overcome barriers to early insulin use—are also needed. Furthermore, the role of technological innovations such as CGM, insulin pumps, and automated insulin delivery systems in early-stage T2DM should be systematically assessed.

## 5 Conclusion

This systematic review demonstrates that early insulin initiation in type 2 diabetes improves short-term glycemic control and may support preservation of β-cell function. However, differences in study design and outcome reporting limit the strength of conclusions regarding long-term safety and cardiovascular outcomes.

Establishing a uniform definition for early insulin initiation, along with clear inclusion criteria for study populations, is essential for advancing research in this field. Maintaining uniformity in glycemic control metrics, including standardized HbA1c thresholds and secondary indicators such as HOMA-IR and C-peptide levels, would enhance the robustness of subsequent research. Standardizing the reporting of cardiovascular and safety outcomes, such as major adverse cardiovascular events (MACE), weight changes, and hypoglycemia incidence, would enable a more precise evaluation of the long-term implications of insulin therapy. Conducting extended follow-up studies is also essential to better understand the potential risks and metabolic benefits over time.

In clinical practice, early insulin initiation should be considered in patients with significant hyperglycemia, rapid disease progression, or inadequate response to oral therapies, particularly when β-cell function is still preserved. Careful patient selection and monitoring are essential to balance glycemic benefits against risks such as hypoglycemia and weight gain.

## Data Availability

The original contributions presented in the study are included in the article/**Supplementary Material**. Further inquiries can be directed to the corresponding author.
