# Supplementary material for: Early insulin initiation in type 2 diabetes: efficacy, safety, and clinical implications: a systematic review with narrative synthesis
Source: Front Endocrinol (Lausanne). 2026 Aug 31;17:1843227. doi: 10.3389/fendo.2026.1843227 (PMC13572116; doi:10.3389/fendo.2026.1843227)
Supplement: Supplementary file 2 [file DataSheet2.pdf]

Search: **((((((((early) AND (initiation)) AND (insulin therapy)) OR (insulin treatment)) OR (basal insulin)) AND (type 2 diabetes mellitus)) AND (glycemic control)) AND (safety profile)) OR (adverse effects)** Filters: **Exclude preprints, from 2010 - 2025**

(((("early"[All Fields] AND ("initial"[All Fields] OR "initially"[All Fields] OR "initials"[All Fields] OR "initiate"[All Fields] OR "initiated"[All Fields] OR "initiates"[All Fields] OR "initiating"[All Fields] OR "initiation"[All Fields] OR "initiations"[All Fields] OR "initiator"[All Fields] OR "initiators"[All Fields]) AND (("insulin"[MeSH Terms] OR "insulin"[All Fields] OR "insulin s"[All Fields] OR "insuline"[All Fields] OR "insulinic"[All Fields] OR "insulinization"[All Fields] OR "insulinized"[All Fields] OR "insulins"[MeSH Terms] OR "insulins"[All Fields]) AND ("therapeutics"[MeSH Terms] OR "therapeutics"[All Fields] OR "therapies"[All Fields] OR "therapy"[MeSH Subheading] OR "therapy"[All Fields] OR "therapy s"[All Fields] OR "therapys"[All Fields]))) OR (("insulin"[MeSH Terms] OR "insulin"[All Fields] OR "insulin s"[All Fields] OR "insuline"[All Fields] OR "insulinic"[All Fields] OR "insulinization"[All Fields] OR "insulinized"[All Fields] OR "insulins"[MeSH Terms] OR "insulins"[All Fields]) AND ("therapeutics"[MeSH Terms] OR "therapeutics"[All Fields] OR "treatments"[All Fields] OR "therapy"[MeSH Subheading] OR "therapy"[All Fields] OR "treatment"[All Fields] OR "treatment s"[All Fields])) OR (("basal"[All Fields] OR "basale"[All Fields] OR "basally"[All Fields] OR "basals"[All Fields]) AND ("insulin"[MeSH Terms] OR "insulin"[All Fields] OR "insulin s"[All Fields] OR "insuline"[All Fields] OR "insulinic"[All Fields] OR "insulinization"[All Fields] OR "insulinized"[All Fields] OR "insulins"[MeSH Terms] OR "insulins"[All Fields]))) AND ("diabetes mellitus, type 2"[MeSH Terms] OR "type 2 diabetes mellitus"[All Fields]) AND ("glycemic control"[MeSH Terms] OR ("glycemic"[All Fields] AND "control"[All Fields]) OR "glycemic control"[All Fields]) AND (("safety"[MeSH Terms] OR "safety"[All Fields] OR "safeties"[All Fields]) AND ("profile"[All Fields] OR "profiled"[All Fields] OR "profiler"[All Fields] OR "profilers"[All Fields] OR "profiles"[All Fields] OR "profiling"[All Fields] OR "profilings"[All Fields]))) OR ("adverse effects"[MeSH Subheading] OR ("adverse"[All Fields] AND "effects"[All Fields]) OR "adverse effects"[All Fields])) AND ((excludepreprints[Filter]) AND (2010:2025[pdat]))

Translations

initiation: "initial"[All Fields] OR "initially"[All Fields] OR "initials"[All Fields] OR "initiate"[All Fields] OR "initiated"[All Fields] OR "initiates"[All Fields] OR "initiating"[All Fields] OR "initiation"[All Fields] OR "initiations"[All Fields] OR "initiator"[All Fields] OR "initiators"[All Fields]

insulin: "insulin"[MeSH Terms] OR "insulin"[All Fields] OR "insulin's"[All Fields] OR "insuline"[All Fields] OR "insulinic"[All Fields] OR "insulinization"[All Fields] OR "insulinized"[All Fields] OR "insulins"[MeSH Terms] OR "insulins"[All Fields]
therapy: "therapeutics"[MeSH Terms] OR "therapeutics"[All Fields] OR "therapies"[All Fields] OR "therapy"[Subheading] OR "therapy"[All Fields] OR "therapy's"[All Fields] OR "therapys"[All Fields]
insulin: "insulin"[MeSH Terms] OR "insulin"[All Fields] OR "insulin's"[All Fields] OR "insuline"[All Fields] OR "insulinic"[All Fields] OR "insulinization"[All Fields] OR "insulinized"[All Fields] OR "insulins"[MeSH Terms] OR "insulins"[All Fields]
treatment: "therapeutics"[MeSH Terms] OR "therapeutics"[All Fields] OR "treatments"[All Fields] OR "therapy"[Subheading] OR "therapy"[All Fields] OR "treatment"[All Fields] OR "treatment's"[All Fields]
basal: "basal"[All Fields] OR "basale"[All Fields] OR "basally"[All Fields] OR "basals"[All Fields]
insulin: "insulin"[MeSH Terms] OR "insulin"[All Fields] OR "insulin's"[All Fields] OR "insuline"[All Fields] OR "insulinic"[All Fields] OR "insulinization"[All Fields] OR "insulinized"[All Fields] OR "insulins"[MeSH Terms] OR "insulins"[All Fields]
type 2 diabetes mellitus: "diabetes mellitus, type 2"[MeSH Terms] OR "type 2 diabetes mellitus"[All Fields]
glycemic control: "glycemic control"[MeSH Terms] OR ("glycemic"[All Fields] AND "control"[All Fields]) OR "glycemic control"[All Fields]
safety: "safety"[MeSH Terms] OR "safety"[All Fields] OR "safeties"[All Fields]
profile: "profile"[All Fields] OR "profiled"[All Fields] OR "profiler"[All Fields] OR "profilers"[All Fields] OR "profiles"[All Fields] OR "profiling"[All Fields] OR "profilings"[All Fields]
adverse effects: "adverse effects"[Subheading] OR ("adverse"[All Fields] AND "effects"[All Fields]) OR "adverse effects"[All Fields]
