# Supplementary material for: Early insulin initiation in type 2 diabetes: efficacy, safety, and clinical implications: a systematic review with narrative synthesis
Source: Front Endocrinol (Lausanne). 2026 Aug 31;17:1843227. doi: 10.3389/fendo.2026.1843227 (PMC13572116; doi:10.3389/fendo.2026.1843227)
Supplement: Supplementary file 3 [file DataSheet3.pdf]

Supplementary table 2: Research Questions and Corresponding Extracted Variables for Early Insulin Initiation in Type 2 Diabetes Mellitus

Research Question / Objective	Specific Extracted Variable(s)
What is the efficacy of early insulin initiation in T2DM?	- Change in HbA1c (glycated hemoglobin)
	- Fasting blood glucose (FBG)
	- self-monitored glucose values
Does early insulin preserve β-cell function?	- β-cell function indices (e.g., HOMA-β, AIR, C-peptide, ISSI-2, insulinogenic index, AUC)
Is early insulin initiation safe compared to delayed/other therapies?	- Incidence of hypoglycemia (overall and severe episodes)
	- Change of BMI & weight gain
What are the effects of early insulin initiation in T2DM on long-term complications?	- Cardiovascular event rate (macrovascular complications)
	- Microvascular complications (retinopathy, nephropathy, neuropathy)
	- All-cause mortality
What are patterns/barriers to early insulin initiation?	- Rates/reasons of patient non-adherence<br>- Adverse event frequency (e.g., hypoglycemia, weight gain as barriers)
	Adverse event frequency (macrovascular complications, all-cause mortality)
	Therapeutic misconception
	Health care resource utilization & cost effect
What study characteristics might moderate the effects observed?	- Study design type (RCT, observational)
	- Sample size, follow-up duration
	- Comparator therapy (e.g., oral agents, delayed insulin)
