# Supplementary material for: Early insulin initiation in type 2 diabetes: efficacy, safety, and clinical implications: a systematic review with narrative synthesis
Source: Front Endocrinol (Lausanne). 2026 Aug 31;17:1843227. doi: 10.3389/fendo.2026.1843227 (PMC13572116; doi:10.3389/fendo.2026.1843227)
Supplement: Supplementary file 4 [file DataSheet4.pdf]

Supplementary table 3: The excluded studies

Study	Reason for exclusion
(Reach et al., 2013)[26]	Different objectives: intrinsic patient characteristics and extrinsic environmental factors predicting prescription and use and, more specifically, early initiation
(Turner et al., 1976)[27]	Publication year
(Garvey et al., 1985) [28]	Publication year
(Wallia et al., 2014)[29]	Study design: Review article
(Abdelgani et al, 2022)[30]	Restricted access
(Sharma et al., 2022)[31]	Different objectives: Cluster analysis of cardiovascular phenotypes in patients with type 2 diabetes and established atherosclerotic cardiovascular disease
(Birkeland et al., 2019) [32]	Different outcomes: The association of basal insulin treatment versus standard care with outcomes in anti-GAD positive and negative subjects
(Landgraf et al., 2022)[33]	Study design: Systematic review
(Hanefeld et al., 2013)[34]	Study design: Review article
(Bolli et al 2015) [35]	Different inclusion criteria; the duration of diabetes more than 5years
Meneghini et.al., , 2010) [36]	
(Swinnen et al 2010) [37]	
(Hanefeld et al 2012) [38]	
