# Supplementary material for: Early insulin initiation in type 2 diabetes: efficacy, safety, and clinical implications: a systematic review with narrative synthesis
Source: Front Endocrinol (Lausanne). 2026 Aug 31;17:1843227. doi: 10.3389/fendo.2026.1843227 (PMC13572116; doi:10.3389/fendo.2026.1843227)
Supplement: Supplementary file 5 [file DataSheet5.pdf]

JBI Checklist for Cohort studies

JBI checklist questions	Chon *et al*., 2010	(Jeon *et al*., 2022)	Nyström et al 2019)	Bailey et al 2019))	Levin et al 2016)	Xie L et al 2011	Karacaer et al 2021
1. Were the two groups similar and recruited from the same population?	Yes	Yes	Yes	Yes	Yes	Yes	Yes
2. Were the exposures measured similarly to assign people to both exposed and unexposed groups?	Yes	Yes	Yes	Yes	Yes	No	Yes
3. Was the exposure measured in a valid and reliable way?	Yes	Yes	Yes	Yes	Yes	Yes	Yes
4. Were confounding factors identified?	Yes	Yes	No	Yes	Yes	Yes	Unclear
5. Were strategies to deal with confounding factors stated?	No	NO	No	Yes	Yes	Yes	No
6. Were the groups/participants free of the outcome at the start of the study (or at the moment of exposure)?	Yes	Unclear	Unclear	Unclear	Yes	No	Unclear
7. Were the outcomes measured in a valid and reliable way?	Yes	Yes	Yes	Yes	Yes	Yes	Yes
8. Was the follow up time reported and sufficient to be long enough for outcomes to occur?	Yes	Yes	Yes	No (short)	Yes	Yes	No
9. Was follow up complete, and if not, were the reasons to loss to follow up described and explored?	Yes	Yes	Yes	Unclear	Yes	No	Yes
10. Were strategies to address incomplete follow up utilized?	No	No	No	No	Unclear	No	Unclear
11. Was appropriate statistical analysis used?	Yes	Yes	Yes	Yes	Yes	Yes	Yes

JBI CRITICAL APPRAISAL CHECKLIST FOR COHORT STUDIES

Reviewer_____ ____Date________________________________

Author____Year_________ Record Number_________

	Yes	No	Unclear	Not applicable
1. Were the two groups similar and recruited from the same population?	☐	☐	☐	☐
2. Were the exposures measured similarly to assign people to both exposed and unexposed groups?	☐	☐	☐	☐
3. Was the exposure measured in a valid and reliable way?	☐	☐	☐	☐
4. Were confounding factors identified?	☐	☐	☐	☐
5. Were strategies to deal with confounding factors stated?	☐	☐	☐	☐
6. Were the groups/participants free of the outcome at the start of the study (or at the moment of exposure)?	☐	☐	☐	☐
7. Were the outcomes measured in a valid and reliable way?	☐	☐	☐	☐
8. Was the follow up time reported and sufficient to be long enough for outcomes to occur?	☐	☐	☐	☐

9. Was follow up complete, and if not, were the reasons to loss to follow up described and explored? ☐ ☐ ☐ ☐

10. Were strategies to address incomplete follow up utilized? ☐ ☐ ☐ ☐

11. Was appropriate statistical analysis used? ☐ ☐ ☐ ☐

Overall appraisal: Include ☐ Exclude ☐ Seek further info ☐

Comments (Including reason for exclusion)

____

____
