# Supplementary material for: Early insulin initiation in type 2 diabetes: efficacy, safety, and clinical implications: a systematic review with narrative synthesis
Source: Front Endocrinol (Lausanne). 2026 Aug 31;17:1843227. doi: 10.3389/fendo.2026.1843227 (PMC13572116; doi:10.3389/fendo.2026.1843227)
Supplement: Supplementary file 6 [file DataSheet6.pdf]

Risk of bias for RCT

Author	selection bias		Blinding of participants & Investigators	Attrition Bias	Reporting bias	Others
	Allocation concealment	Random sequence generation				
(Hu Y et al 2011)	√	Unclear	×	√	√	-
Harrison et al 2012)	√	√	√	×	√	-
(Gerstein et al 2012)	√	√	×	√	√	-
Zeng et al 2012)	√	√	×	√	√	Short-term
Pistrosch et al 2013	Unclear	Unclear	×	The dropout rate was identical between metformin (n = 5)- and glargine (n = 6	√	small sample size
Wang et al 2019	×	×	×	√	√	small sample size
Retnakaran et al 2021	√	√	×	√	√	beta-cell function and insulin sensitivity/resistance were assessed with OGTT-based surrogate indices rather than clamp studies<br><br>Conflict of interest
(Pigeyre *et al.*, 2022)	√	√	×	√		
Retnakaran et al 2023	Unclear	√	Unclear	√	√	modest sample size
